# Influence of Polyphosphoric Acid on the Consistency and Composition of Formulated Bitumen: Standard Characterization and NMR Insights

**DOI:** 10.1155/2016/2915467

**Published:** 2016-08-08

**Authors:** Catarina Varanda, Inês Portugal, Jorge Ribeiro, Artur M. S. Silva, Carlos M. Silva

**Affiliations:** ^1^Department of Chemistry, CICECO-Aveiro Institute of Materials, University of Aveiro, 3810-193 Aveiro, Portugal; ^2^Galp Energia, Refinaria de Matosinhos, Rua Belchior Robles, 4450-802 Leça da Palmeira, Portugal; ^3^Department of Chemistry, QOPNA, University of Aveiro, 3810-193 Aveiro, Portugal

## Abstract

Over the recent years, bitumen modification with polymers, acids, or mineral fillers has gained relevance to adjust its performance properties. This work reports the use of polyphosphoric acid (PPA) for the modification of formulated bitumen. With this objective, an in-depth literature review on PPA modification was firstly performed. Subsequently, five individual refinery components were selected for the preparation of bitumen blends, namely, asphaltic residue, vacuum residue, and three lube oils extracts. Seven binary/ternary bitumen blends were prepared and then treated with PPA. Afterwards, the five components and the unmodified and PPA-modified bitumen were characterized by standard methods (penetration, softening point, and penetration index), SARA analysis, elemental analysis, and ^31^P and ^1^H nuclear magnetic resonance (NMR) spectroscopy. The results evidenced higher asphaltenes and lower saturates/resins contents in PPA-modified bitumen. The NMR data suggest that the paraffinic chains became longer, the content of condensed aromatics increased, more substituted aromatic structures appeared, and *α*-hydrogen in aromatic structures diminished. These findings disclosed the improved consistency and oxidation stability of PPA-modified bitumen blends.

## 1. Introduction

The European Committee for Standardization defines bitumen as a virtually nonvolatile, adhesive, and waterproofing dark brown viscous organic material derived from crude oil or present in natural asphalt [1]. Due to its binding and hydrophobic properties, bitumen is commonly used for the construction of pavements and waterproof systems [2, 3]. Bitumen properties are related to its composition, which in turn depends strongly on the type and origin of the crude oil. However, the precise identification of the molecular composition and structure of bitumen is almost impossible; therefore, they are often characterized by fractionation techniques [3, 4]. For instance, the SARA method is based on sequential chromatographic separation of bitumen components into four generic groups according to their polarity and solubility, namely, saturates (S), aromatics (A), resins (R), and asphaltenes (A) [3, 5, 6]. Commonly, the saturates fraction contains linear and branched alkanes eluted with a paraffinic solvent (e.g.,* n*-hexane), the aromatics fraction is constituted mainly by alkylated C5 and C6 cyclic structures eluted with a moderately polar solvent (e.g., toluene), the resins are alkylated and cycloalkylated structures with 2-3 aromatic rings (e.g., eluted with tetrahydrofuran), and asphaltenes are heavier aromatic polycyclic structures [3]. Moreover, bitumen contains heteroatoms (such as sulfur, oxygen, and nitrogen) normally embedded in the molecular structure of the aromatic rings [4, 7].

The structure of bitumen is controversial but the colloidal model, proposed by Rosinger [8] though generally attributed to Nellensteyn [9], still prevails, especially for explaining the interactions between modifiers and bitumen fractions [6, 10]. According to this model, bitumen is considered as a suspension of asphaltene micelles peptized by resins, dispersed in an oily medium constituted by saturates and aromatics [3, 10–12]. Depending on the relative contents of these fractions, bitumen exhibits different rheological properties used for its classification into three distinct types, specifically: “sol” (viscous) bitumen which has a colloidal structure constituted by noninteracting micelles and behaves as Newtonian fluid; “gel” (elastic) bitumen which is characterized by a three-dimensional colloidal structure and high resilience; and “sol-gel” (viscoelastic) bitumen which exhibits an intermediate behavior with elastic effects in the initial stages of deformation and a colloidal structure characterized by the presence of supermicelles [13, 14].

Over the recent years, bitumen has become a highly technical material with modifiers (e.g., polymers, acids, and mineral fillers) gaining relevance in the control of the performance properties of modified bitumen [3, 15]. Polyphosphoric acid (PPA) is an example of a reactive modifier that can be used by itself or in conjugation with a suitable polymer, in this case, with considerable economic benefits [16]. In general, the concentrations of PPA in bitumen are in the range from 0.2 to 1.2 wt.% [7] with an optimum at 1.0 wt.% [17]. Typically, commercial PPA is a mixture of phosphoric acid (H_3_PO_4_), pyrophosphoric acid (H_4_P_2_O_7_), triphosphoric acid (H_5_P_3_O_10_), and higher oligomers [18].

Bitumen refined from a specific crude oil has characteristic properties related to its consistency, namely, softening point and penetration value at 25°C (expressed in dmm) which is the base for the European grading system [19]. These properties can be shifted up or down in the refining process or by posterior blending with different grade bitumen or an additive (e.g., a polymer, PPA, or a combination of both) [20]. Bitumen modification with a polymer or with PPA stiffens the product at higher temperatures, improving the resistance to permanent deformation, without negative effects at low temperatures (i.e., between −30 and 5°C) [18]. It is noteworthy that PPA has been reported to improve the behavior of modified bitumen also at low temperature (≤5°C) [21] and to impart antioxidant properties [18].

In this work, a detailed review of issued patents and scientific literature on the performance of straight-run bitumen (i.e., obtained directly from crude oil distillation) modified with PPA has been accomplished and is presented in the Appendix. On the other hand, since the influence of PPA on formulated bitumen is not reported in the scientific literature, this is the main focus of this paper. It has been studied using phosphorous (^31^P) and proton (^1^H) nuclear magnetic resonance spectroscopy to evaluate structural modifications, SARA and elemental analyses to evaluate chemical transformations, and standard grading tests (softening point, penetration value, and penetration index) for performance evaluation.

## 2. Bitumen Modification with PPA and Its Characterization

**Figure 1:**
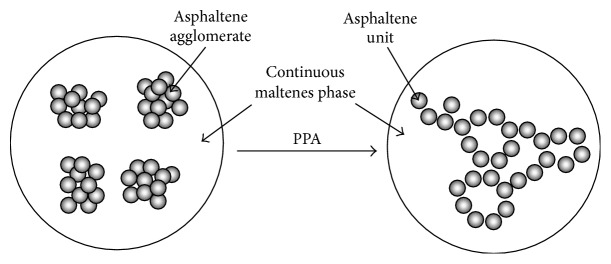
Influence of PPA on the colloidal structure of bitumen (adapted from [17]).

The interaction of PPA with bitumen model compounds was studied by several authors, as summarized in the Appendix. From these studies, it became clear that PPA modification follows different mechanisms depending on the chemical composition of bitumen which is strongly related to the crude oil geographical source. Overall, the reactivity of PPA increases with the polarity of the asphaltene fraction because it enhances PPA dissociation (into PPA^−^ and H^+^) [3] disrupting the hydrogen bond network formed within the agglomerates of asphaltene micelles. Consequently, the molecular weight of the asphaltene fraction is lowered and the distribution of asphaltenes in the remaining fractions (i.e., saturates, aromatics, and resins, usually called maltene phase) is improved, shifting the bitumen towards a more elastic gel-type structure, as represented schematically in Figure 1 [13, 17].

There is a direct relationship between the chemical composition and colloidal structure of bitumen and its performance [13]. Therefore, bitumen is often characterized by related parameters, such as the index of colloidal instability (defined as the ratio of the sum of asphaltenes and saturates to the sum of aromatics and resins), the colloidal indices Ip (i.e., resins-to-asphaltenes ratio) and Is (i.e., ratio of asphaltenes to maltenes), the penetration index (PI), and other rheological measurements.

Several rheological studies of PPA-modified bitumen focused on its performance and stability. For instance, Giavarini et al. [13] reported that PPA-modified bitumen exhibits higher penetration index (PI) and thus improved thermal susceptibility in comparison to unmodified bitumen. Giavarini et al. [16] and Bonemazzi and Giavarini [14] performed rheological studies with polymer-modified bitumen and proved that PPA changes the bitumen structure towards gel type, thus improving the stabilization interactions between the polymer and the bitumen components. Edwards et al. [22] performed rheological tests at low temperature (−25°C) and established that bitumen modification is influenced by the amount and type (grade) of PPA used and also by the composition of bitumen. Baldino et al. [21] validated the capacity of PPA to decrease the glass transition temperature (Tg) and to increase the stiffness of modified bitumen.

Another approach is to use spectroscopic techniques to detect specific functional groups in highly complex hydrocarbon mixtures such as bitumen [23–26]. In particular, solution-state ^1^H and ^13^C nuclear magnetic resonance (NMR) spectroscopy can be used to identify and quantify the aromatic and aliphatic moieties. This is of utmost importance to understand the molecular interactions between the SARA fractions of bitumen and to explain the influence of modifiers on bitumen performance [27]. For instance, Michon et al. [28, 29] used quantitative ^13^C NMR data coupled with molecular weight distribution data to estimate structural parameters of bitumen such as, for example, aromaticity and the average number of naphthenic and aromatic rings per molecule. Their final purpose was to define a bitumen “fingerprint” [28], particularly for the aromatic part, in order to propose a mechanism for bitumen oxidation [29]. The chemical transformations associated with bitumen ageing were investigated by Siddiqui and Ali [30] and Siddiqui [31] using ^1^H and ^13^C NMR. Curiously, Molina et al. [23] developed a very fast and reliable method that correlates the area of ^1^H NMR signals of petroleum residues to their SARA fractions and to some physicochemical properties (e.g., density and S, N, and wax content). Gentile et al. [32] related different ^1^H NMR spin-spin relaxation times (evaluated by inverse Laplace transform) to different macroaggregates in PPA-modified bitumen. Moreover, bitumen modification with PPA can be analyzed by ^31^P NMR, a powerful tool to evaluate the structure of phosphorus-containing compounds.

## 3. Experimental Procedures

### 3.1. Preparation of Bitumen Blends Modified with PPA

The bitumen blends (penetration grade 70/100) used in this work contained asphaltic residue (AR), vacuum residue (VR), and aromatic extracts SN1, SN2, or SN3, in the proportions presented in Table 1. These bitumen blends were provided by a Portuguese refinery.

Bitumen modification with PPA was performed in a glass reactor, equipped with a mechanical stirrer and a temperature controller. Bitumen blends (1000 ± 0.01 g) were heated to approximately 135°C and then PPA grade 105% (supplied by Innophos, Cranbury, USA) (8.00 ± 0.01 g) was added, which corresponds to 0.8 wt.%. The mixture was allowed to react for 30 minutes at 135°C with constant stirring (ca. 245 rpm).

### 3.2. Characterization of Bitumen Components and Blends

The SARA fractions of the individual components and the seven bitumen blends (Bit_*j*_,* j* = 1–7) were analyzed according to the International Standard method IP-469 using Iatroscan MK-6 TLC-FID instrument (Iatron Laboratories Inc.) [33]. Data acquisition was performed with the DataApex Clarity Lite software, version 3. Each sample was analyzed in triplicate and the results (peak areas) were averaged and considered as the weight percentage of the corresponding SARA fractions.

Elemental analysis of carbon, hydrogen, and nitrogen in bitumen samples was performed in a Leco TruSpec® instrument (628 Series) following the standard procedure ASTM D5291 [34]. Sulfur content was determined by energy-dispersive X-ray fluorescence (EDXRF) in Oxford Instruments Lab-X3500, following the International Standard method IP 336 [35]. The analyses were performed using three replicated samples.

For NMR analysis, the bitumen samples (0.20 ± 0.01 g) were dissolved in deuteriochloroform (CDCl_3_, Sigma-Aldrich, 99.8 atom% D) (1 mL) and analyzed in triplicate. Solution-state ^1^H NMR measurements were obtained in a Bruker Avance 300 spectrometer (operating at 300.13 MHz) under the following conditions: spectral window of 6887 Hz, with a 30° pulse width (3.63 *μ*s), acquisition time 2.4 s, relaxation delay 1 s, 32768 data points, and 128 scans. Solution-state ^31^P NMR spectra were acquired at room temperature in a Bruker Avance 300 spectrometer at a nominal frequency of 121.5 MHz, over a period of 7 minutes, using a pulse repetition rate of 1 s. Chemical shifts were referenced to orthophosphoric acid at 0 ppm.

Bitumen penetration (Pen, expressed in dmm) at 25°C and the ring and ball softening point (SP, in °C) were measured by standard methods [19]. The penetration index (PI) was calculated by [36]

(1) $$\begin{array}{c} \text{PI = }\frac{\text{12.04 }\left(\text{SP}-T_{P}\right)-\text{300}\left(\text{2.903}-\log\left(\text{Pen}\right)\right)}{\text{0.602 }\left(\text{SP}-T_{P}\right)\text{ + 30}\left(\text{2.903}-\log\left(\text{Pen}\right)\right)}, \end{array}$$

where *T*_*P*_ (°C) is the temperature used to measure Pen.

## 4. Results and Discussion

### 4.1. SARA and Elemental Analysis of Bitumen Components and Blends

The SARA fractions of the individual bitumen components and of the seven bitumen blends before and after PPA modification are presented in Tables 2 and 3, respectively.  Table 3 also presents the elemental analysis of bitumen before PPA modification.

The binary blends (Bit1, Bit2, and Bit3) prepared with asphaltic residue (AR) and one of the extracts (SN1, SN2, or SN3) present similar contents of asphaltenes and resins (within experimental error). In contrast, the concentration of saturates and aromatics differs for the blends prepared with extracts SN1 and SN2 (i.e., Bit1 and Bit2, resp.) and with SN3 extract (i.e., Bit3). For the former, saturates were not detected and the aromatic content is naturally higher (ca. 71 wt.% versus 65.5 wt.% for Bit3) due to the nature of the extracts (see Table 2). However, the differences in elemental composition of the three bitumen blends are negligible (see Table 3).

It is noteworthy that the SARA fractions for binary blend Bit3 (78.39 wt.% AR + 21.61 wt.% SN3 extract) are similar to those for Bit7, a binary blend prepared with asphaltic and vacuum residues (30 and 70 wt.%, resp.; see Table 1). The most significant difference is the high content of saturates (3.0 wt.%, Table 3) in Bit7 which can be related to its higher content of vacuum residue (VR). In fact, VR contains 5.5 wt.% of saturates whereas AR, the main component of the other blends, contains no saturates (see Table 2). Again, there are no significant differences for the elemental chemical composition of Bit7 in comparison to the other bitumen samples.

The results for the ternary blends (Bit4–Bit6) prepared with AR, VR, and one of the extracts are quite similar (within experimental error) in what concerns the SARA fractions and elemental composition (Table 3). Overall, these results are consistent with the origin of the blends as they were all prepared with components derived from Arabian light crude oil.

It is worth noting that the elemental analysis of formulated bitumen after PPA addition is not reported in Table 3, as the results were statistically equivalent. In fact, from the literature review, it became clear that PPA modification follows different mechanisms, like redox reactions and disruption of hydrogen bond networks, and none of these mechanisms are expected to change the overall elemental composition of PPA-modified bitumen.

A final comment concerning the absence of saturates in Bit1 and Bit2 is in order. In fact, these blends were prepared with extracts SN1 and SN2 which contain ca. 4 wt.% of saturates (Table 2) and thus the absence of saturates is puzzling. A possible explanation is that the content of saturates is below the TLC-FID apparatus detection limit (detection limit 0.8%).

The influence of PPA on bitumen composition (SARA fractions) can be visualized in Table 3. In general, PPA modification decreases the content of saturates and resins and increases the fraction of asphaltenes in agreement with the literature [13, 37]. The higher content of asphaltenes in PPA-modified bitumen explains the experimental results (Table 4), namely, higher softening points (ca. 1–3°C increment), lower penetration values (ca. 10 dmm), and, consequently, an improvement of the penetration index (PI) in accordance with the results reported by Oyekunle [38]. The higher values of PI indicate a change in the colloidal structure of PPA-modified bitumen blends which is expected to improve their thermal susceptibility [13].

The effect of PPA on bitumen modification has been associated with redox reactions involving polycondensed aromatic structures that act as reducing agents for phosphoric acid oligomers (HPO_3_)_*n*_, which are therefore oxidized [13]. As a result, one would expect the conversion of part of the resins fraction into asphaltenes and the conversion of the saturates and aromatics fractions into resins [13]. However, the increase of the aromatics fraction observed in this work, after PPA addition, suggests that part of the resins were converted to asphaltenes and part were converted to aromatics.

### 4.2. Analysis of PPA and Modified Bitumen by ^31^P NMR Spectroscopy

**Figure 2:**
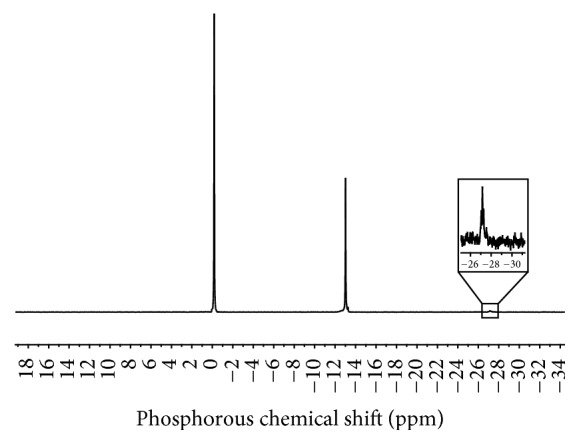
^31^P NMR spectrum of the PPA (105% grade) used for bitumen modification.

The ^31^P NMR spectrum of the PPA used in this work is presented in Figure 2. According to the literature [37, 39], the resonance peak at 0 ppm is attributed to phosphorus in H_3_PO_4_, a smaller peak at −13 ppm is assigned to phosphorus in end groups of PPA chains, and a much smaller peak around −26 ppm is attributed to phosphorus in the middle groups of PPA chains.

**Figure 3:**
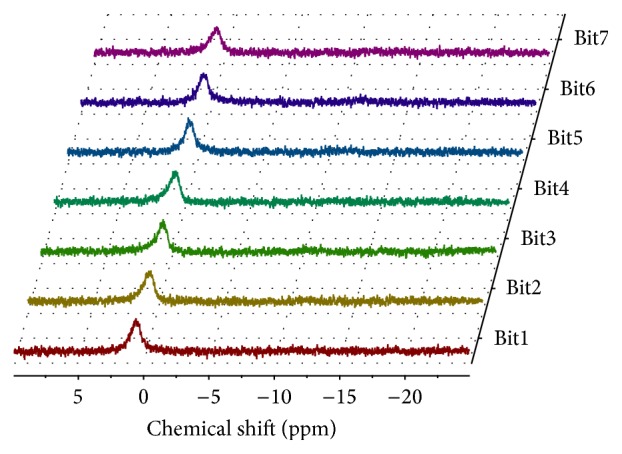
^31^P NMR spectra for PPA-modified bitumen (Bit1–Bit7).

The PPA-modified bitumen blends were also analyzed by ^31^P NMR spectroscopy, at room temperature. The spectra for the seven blends (see Figure 3) are very similar revealing a broad signal approximately at 1 ppm. This resonance peak, located in the vicinity of the phosphorous resonance peak in H_3_PO_4_, was assigned to hydrophobic interactions between phosphorous and polycondensed aromatic structures typically present in asphaltenes [23]. The absence of resonance peaks assigned to phosphorus in phosphate chains (end and middle groups) suggests that phosphoric esters were not formed during bitumen modification with PPA, in agreement with the results of Miknis and Thomas [39] and in opposition to the earlier hypothesis of Orange et al. [40]. Furthermore, these results indicate that PPA is hydrolyzed completely back to H_3_PO_4_, probably due to the presence of residual water in bitumen [39].

The broader spectral peaks for PPA-modified bitumen blends (Figure 3), in comparison with PPA (Figure 2), are probably due to higher viscosity of the PPA bitumen blends or to the formation of charge transfer complexes between phosphorous and the asphaltene polycondensed aromatic structures [39, 41]. Another hypothesis is that small amounts of paramagnetic impurities typically present in bitumen (e.g., vanadium and nickel) may decrease the relaxation time and, consequently, increase peak broadening due to the Heisenberg uncertainty principle [42].

### 4.3. Analysis of Unmodified and PPA-Modified Bitumen by ^1^H NMR Spectroscopy

The ^1^H NMR results for unmodified and PPA-modified bitumen blends are presented in Table 5. The spectra were divided into 12 unequal segments corresponding to well-defined chemical shifts associated with specific groups (i.e., hydrogen types), though minor overlapping may occur [23]. Peak integration in the selected segments generated, after normalization, the relative content of the various proton types (Table 5). The mean values were obtained by averaging the results from the spectra of three aliquots of the same bitumen formulation.

**Figure 4:**
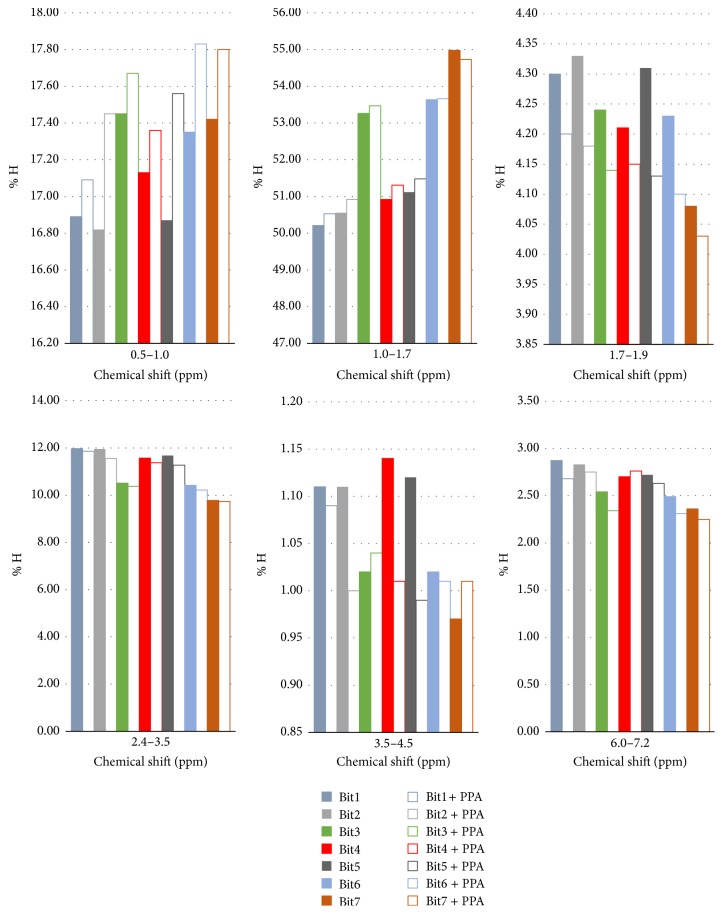
Most significant results from the ^1^H NMR spectra of bitumen blends before and after PPA addition. The types of hydrogen associated with the chemical shift regions are listed in Table 5.

Some conclusions may be drawn from the ^1^H NMR results (Table 5) more explicitly presented in Figure 4. In general, the content of aliphatic hydrogen (segments 1 and 2, Table 5) in PPA-modified bitumen is different from that in the parent unmodified bitumen. The signals in the segments associated with the branching (0.5–1.0 ppm) and the length (1.0–1.7 ppm) of paraffinic chains are systematically higher, though rather similar. The signals in segment 3 (1.7–1.9 ppm), that is, in the region of chemical shifts associated with sulfur adjacent hydrogen atoms (1.8–3.0 ppm) [27], decrease after PPA modification, thus suggesting more substituted aromatic structures.

The slightly lower signals in segment 6 (2.4–3.5 ppm), Figure 4, which are associated with the chemical shifts of *α*-hydrogen (*α*-CH, *α*-CH_2_) indicate the occurrence of oxidation reactions, thus explaining the improved stability of PPA-modified bitumen [27]. The signals associated with bridging hydrogen atoms (segment 7, 3.5–4.5 ppm) and hydrogen atoms in monoaromatic structures (segment 9, 6.0–7.2 ppm) have both a tendency to decrease after PPA modification (see Figure 4). This might be related to a slight increase in the content of hydrogen atoms associated with polyaromatic structures (segments 10, 11, and 12, in Table 5), suggesting an increase in content of condensed structures as reported in the literature [13].

A final note about the residual presence of olefins, detected in the region 4.5–6.0 ppm (segment 8, Table 5). In fact, since bitumen is allegedly free of olefins, due to the process conditions in the refinery, its presence can be attributed to cracking reactions eventually occurring during NMR sample preparation (recall that bitumen reheating was necessary to facilitate sample handling) [54].

## 5. Conclusions

Binary and ternary bitumen blends formulated with asphaltic residue, vacuum residue, and aromatic- extracts derived from refined Arabian crude oil were analyzed before and after modification with polyphosphoric acid (PPA). SARA analysis revealed that, in general, PPA modification increased the fraction of asphaltenes in bitumen and decreased the saturates and resins fractions abundance. These changes improved the performance properties, namely, the penetration index (PI), which indicates higher thermal stability of PPA-modified bitumen blends.

With respect to the mechanism of PPA actuation, the ^31^P NMR proved that PPA is hydrolyzed back to phosphoric acid and reacts with bitumen, probably in the form of charge transfer complexes with polycondensed aromatic structures. This fact was disclosed from the presence of the single resonance peak assigned to phosphoric acid and the absence of peaks assigned to phosphorus in the end and middle groups of phosphate chains. The PPA disrupts the agglomerates of asphaltene micelles, which promotes the distribution of asphaltenes in the maltene phase, thus increasing the content of the aromatic fraction. Furthermore, part of the resins is converted into the aromatic fraction.

Concerning the ^1^H NMR studies, results suggest that PPA improves the oxidation stability of the bitumen blends and increases the concentration of condensed structures. The former was evidenced by the content drop of *α*-hydrogen (range 2.4–3.5 ppm) after PPA modification, as these hydrogen atoms are associated with oxidation susceptibility.

## Figures and Tables

**Table 1:** Composition (wt.%) of the bitumen formulations.

Blend	Component				
	AR	VR	SN1	SN2	SN3
Bit1	84.80		15.20		
Bit2	78.44			21.56	
Bit3	78.39				21.61
Bit4	77.31	9.99	12.70		
Bit5	70.69	10.02		19.29	
Bit6	72.27	10.06			17.67
Bit7	30.00	70.00			

**Table 2:** SARA analysis of the individual components of bitumen blends.

Component	Saturates	Aromatics	Resins	Asphaltenes
	(wt.%)			
Asphaltic residue (AR)	—	61.6 ± 1.3	21.7 ± 1.5	16.7 ± 1.0
Vacuum residue (VR)	5.5 ± 0.7	63.8 ± 1.7	17.3 ± 1.5	13.4 ± 0.9
SN1 extract	4.1 ± 0.5	92.6 ± 0.9	3.3 ± 0.6	—
SN2 extract	3.6 ± 0.3	92.8 ± 0.6	3.6 ± 0.5	—
SN3 extract	9.9 ± 0.8	84.9 ± 1.2	5.2 ± 0.8	—

**Table 3:** Elemental chemical analyses of unmodified bitumen blends and SARA analysis of unmodified (before PPA) and modified (after PPA) bitumen blends.

Bitumen	Elemental analysis before PPA (wt.%)^a^				SARA analysis before PPA (wt.%)^a^				SARA analysis after PPA (wt.%)^a^			
Sample	**C**	**H**	**N**	**S**	Saturates	Aromatics	Resins	Asphaltenes	Saturates	Aromatics	Resins	Asphaltenes
	(±1.3)	(±0.4)	(±0.10)	(±0.09)								
Bit1	84.7	10.3	0.45	4.74	n.d.	66.4 ± 0.8	18.9 ± 0.7	14.2 ± 0.7	n.d.	71.2 ± 0.9	13.5 ± 1.0	15.2 ± 0.8
Bit2	84.8	10.4	0.42	4.76	n.d.	68.3 ± 1.0	17.8 ± 1.0	13.1 ± 0.9	n.d.	70.7 ± 1.3	13.4 ± 0.9	15.9 ± 1.0
Bit3	84.2	10.6	0.43	4.43	1.9 ± 0.2	66.6 ± 1.4	18.2 ± 0.6	13.1 ± 0.9	1.6 ± 0.2	65.5 ± 1.6	14.9 ± 1.5	14.9 ± 0.7
Bit4	84.6	10.3	0.43	4.66	1.1 ± 0.2	65.8 ± 1.6	18.9 ± 0.8	14.3 ± 1.0	0.7 ± 0.2	68.0 ± 1.1	14.7 ± 1.2	16.6 ± 0.8
Bit5	84.4	10.4	0.43	4.69	1.2 ± 0.2	67.8 ± 0.8	17.8 ± 1.2	13.2 ± 0.8	1.0 ± 0.2	70.5 ± 1.2	13.6 ± 0.8	14.9 ± 0.9
Bit6	84.2	10.6	0.42	4.40	2.2 ± 0.2	65.6 ± 0.9	18.6 ± 0.9	13.6 ± 0.7	1.6 ± 0.2	67.4 ± 0.2	15.6 ± 1.8	15.4 ± 0.8
Bit7	84.8	10.9	0.43	4.35	3.9 ± 0.5	63.1 ± 0.2	18.6 ± 1.2	14.4 ± 0.7	3.0 ± 0.4	66.6 ± 1.7	15.2 ± 1.1	15.1 ± 0.7

^a^Average ± standard deviation; n.d.: not detected.

**Table 4:** Characteristic properties of bitumen blends, before and after PPA modification.

Blend	Before PPA			After PPA		
	Pen (dmm)	SP (°C)	PI (—)	Pen (dmm)	SP (°C)	PI (—)
Bit1	101	43.0	−1.4	90	43.8	−1.5
Bit2	99	43.0	−1.5	88	44.0	−1.5
Bit3	103	43.0	−1.4	90	45.2	−1.1
Bit4	108	42.0	−1.6	88	44.2	−1.4
Bit5	99	43.0	−1.5	95	43.8	−1.3
Bit6	110	42.2	−1.5	86	45.5	−1.1
Bit7	100	43.5	−1.3	91	45.6	−0.9

Pen: penetration at 25°C; SP: ring and ball softening point; PI: penetration index.

**Table 5:** ^1^H NMR results for the bitumen before and after PPA modification.

Segment	Chemical shift (ppm)	Hydrogen type	Bit1	Bit1 + PPA	Bit2	Bit2 + PPA	Bit3	Bit3 + PPA	Bit4	Bit4 + PPA	Bit5	Bit5 + PPA	Bit6	Bit6 + PPA	Bit7	Bit7 + PPA
			%						%							
1	0.5–1.0	*γ* CH_3_, some naphthenic CH and CH_2_	16.89 ± 0.22	17.09 ± 0.12	16.82 ± 0.04	17.45 ± 0.01	17.45 ± 0.18	17.67 ± 0.07	17.13 ± 0.24	17.36 ± 0.13	16.87 ± 0.08	17.56 ± 0.06	17.35 ± 0.10	17.83 ± 0.12	17.42 ± 0.08	17.80 ± 0.19
2	1.0–1.7	*β* CH_2_ and some *β* CH	50.21 ± 0.03	50.53 ± 0.08	50.56 ± 0.11	50.92 ± 0.20	53.26 ± 0.16	53.47 ± 0.16	50.92 ± 0.09	51.31 ± 0.25	51.12 ± 0.16	51.48 ± 0.16	53.63 ± 0.23	53.66 ± 0.06	54.98 ± 0.22	54.73 ± 0.18
3	1.7–1.9	Most CH and CH_2_ in *β* positions	4.30 ± 0.02	4.20 ± 0.02	4.33 ± 0.03	4.18 ± 0.02	4.24 ± 0.06	4.14 ± 0.00	4.21 ± 0.06	4.15 ± 0.04	4.31 ± 0.03	4.13 ± 0.03	4.23 ± 0.05	4.10 ± 0.04	4.08 ± 0.02	4.03 ± 0.06
4	1.9–2.1	*α* CH_3_ in olefins	2.35 ± 0.01	2.27 ± 0.02	2.35 ± 0.03	2.23 ± 0.01	2.29 ± 0.05	2.23 ± 0.01	2.27 ± 0.05	2.24 ± 0.03	2.33 ± 0.02	2.20 ± 0.02	2.28 ± 0.04	2.19 ± 0.03	2.20 ± 0.03	2.16 ± 0.05
5	2.1–2.4	*α* CH_3 _in aromatic carbons	4.19 ± 0.03	4.07 ± 0.02	4.15 ± 0.02	3.97 ± 0.02	3.77 ± 0.04	3.65 ± 0.01	3.99 ± 0.04	3.97 ± 0.04	4.06 ± 0.02	3.89 ± 0.02	3.73 ± 0.01	3.61 ± 0.02	3.52 ± 0.01	3.42 ± 0.01
6	2.4–3.5	*α* CH and CH_2 _in aromatic carbons	11.97 ± 0.09	11.8 ± 0.10	11.95 ± 0.08	11.55 ± 0.06	10.51 ± 0.13	10.38 ± 0.06	11.57 ± 0.11	11.37 ± 0.04	11.68 ± 0.03	11.27 ± 0.07	10.42 ± 0.10	10.22 ± 0.05	9.78 ± 0.07	9.74 ± 0.10
7	3.5–4.5	Bridging CH_2_	1.11 ± 0.04	1.09 ± 0.07	1.11 ± 0.06	1.00 ± 0.03	1.02 ± 0.09	1.04 ± 0.03	1.14 ± 0.07	1.01 ± 0.07	1.12 ± 0.06	0.99 ± 0.04	1.02 ± 0.10	1.01 ± 0.05	0.97 ± 0.08	1.01 ± 0.08
8	4.5–6.0	Olefins	0.07 ± 0.02	0.10 ± 0.03	0.04 ± 0.03	0.07 ± 0.01	0.06 ± 0.06	0.09 ± 0.02	0.11 ± 0.03	0.07 ± 0.03	0.06 ± 0.06	0.06 ± 0.02	0.05 ± 0.08	0.09 ± 0.02	0.04 ± 0.07	0.14 ± 0.09
9	6.0–7.2	Monoaromatics	2.87 ± 0.10	2.68 ± 0.19	2.83 ± 0.17	2.75 ± 0.05	2.54 ± 0.12	2.34 ± 0.10	2.70 ± 0.08	2.76 ± 0.04	2.72 ± 0.10	2.63 ± 0.16	2.49 ± 0.13	2.31 ± 0.19	2.36 ± 0.11	2.25 ± 0.08
10	7.2–8.3	Diaromatics and some tri- and tetra-aromatics	5.23 ± 0.02	5.25 ± 0.05	5.11 ± 0.06	5.10 ± 0.05	4.20 ± 0.02	4.26 ± 0.03	5.11 ± 0.05	4.99 ± 0.08	4.97 ± 0.06	4.98 ± 0.07	4.12 ± 0.06	4.24 ± 0.02	3.97 ± 0.05	4.01 ± 0.08
11	8.3–8.9	Some tri- and tetra-aromatics	0.67 ± 0.01	0.71 ± 0.04	0.64 ± 0.04	0.66 ± 0.03	0.55 ± 0.02	0.61 ± 0.01	0.71 ± 0.03	0.65 ± 0.05	0.64 ± 0.04	0.67 ± 0.04	0.53 ± 0.04	0.61 ± 0.02	0.53 ± 0.03	0.59 ± 0.02
12	8.9–9.3	Some tetra-aromatics	0.12 ± 0.01	0.14 ± 0.02	0.10 ± 0.02	0.11 ± 0.01	0.10 ± 0.01	0.13 ± 0.00	0.14 ± 0.01	0.12 ± 0.02	0.11 ± 0.02	0.12 ± 0.02	0.10 ± 0.02	0.13 ± 0.01	0.10 ± 0.02	0.12 ± 0.01

**Table 6:** Summary of issued patents related to bitumen modification with PPA.

Year	Type	Patent number	Licensing Company
1973	Chemically modified bitumen	US3751278 A	Tosco Lion Inc.
2009		US20090249978A1	Innophos

1991	Polymer-modified bitumen	US5070123 A	Exxon Research & Engineering
1996		US5565510 A	Montell North America Inc.
1996		US5519073 A	Shell Oil Company
1999		US5880185 A	Elf Exploration Production
2000		US6117926 A	Mathy Construction Company
2000		US6031029 A	Ergon
2000		US6136898 A	Marathon Ashland Petroleum
2002		US6414056 A	Exxon Mobil
2007		US20050284333	ICL Performance Products
2008		US7985787	Innophos

2010	Reclaimed asphalt pavement	US8906152	Innophos

2006	Roofing membranes	US7678467 B2	ICL Performance Products
2013		WO2013116637 A1	ICL Performance Products

2004	Crumb rubber modified bitumen	WO2004081098 A1	Eurovia
2011		WO 2011047032 A2	Innophos
2011		WO 2011057085 A2	Innophos

Search criteria. Keywords: (asphalt or bitumen) and (polyphosphoric acid) in title and abstract in Google Patents and Web of Knowledge databases. Accessed in December 2015.

**Table 7:** Literature review about bitumen modification with polyphosphoric acid (PPA).

Year	Bitumen type (source)	PPA, wt.%	Other additives^a^	Aim of PPA modification	Parameters analyzed^b^	Principal observations	Reference
1995	Straight-run bitumen (various undisclosed crude oils)	1-2	—	Study the physical and chemical properties of modified bitumen	Pen; SP; FraassT; ageing (RTFO); MW; asphaltenes; ^1^H, ^13^C, ^31^P NMR; morphological and rheological parameters	(i) PPA modification is similar to mild air-blown modification of bitumen, with lower costs (ii) Higher penetration index and ageing resistance with minor losses for low-temperature behavior (iii) Shifting towards a gel structure with higher content and MW of asphaltenes, due to conversion of aromatics to resins and of resins to asphaltenes and to the formation of asphaltene-phosphorous complexes	[37]

1996	Straight-run bitumen (various undisclosed crude oils)	1–5	Ethylene-propylene copolymers (2–10 wt.%)	Improve storage stability of polymer-modified bitumen	Pen; SP; viscosity; rheological curves; asphaltenes; MW; storage stability; morphology	(i) PPA improves the dispersion of the copolymer in the bitumen, shifting it towards a gel structure with increased content and MW of the asphaltenes fraction (ii) PPA modification imparts higher penetration index, viscosity, and better storage stability at high temperatures (iii) The addition of PPA can reduce copolymer load (by ca. 50%) thus lowering the costs	[16]

1999	Straight-run and visbreaker bitumen (undisclosed source)	1–3	Ethylene-propylene copolymers (2 or 5 wt.%)	Study the colloidal structure of polymer-modified bitumen	Pen; SP; MW; asphaltenes; storage stability; rheological parameters	(i) Similar properties for PPA-modified bitumen and air-blown bitumen, except at low temperature (ii) Improved behavior at high temperature, unchanged behavior at low temperature (Fraass brittle point) (iii) Shifting from sol to gel colloidal structure (iv) Higher penetration index and storage stability	[14]

2000	Vacuum residue (Middle East)	1	—	Improve the rheological and physicochemical properties of vacuum residues	Pen; SP; SARA; ^13^C NMR; elemental analysis; rheological and colloidal parameters	(i) Higher stiffness, improved elasticity and colloidal stability, and lower thermal susceptibility (ii) Shifting towards a gel type structure (related to more aromatic and polycondensed asphaltenes) (iii) Conversion of maltenes to asphaltenes (iv) Formation of asphaltene-phosphorous complexes	[13]

2004	Bitumen blends (Saudi Arabia, Venezuela, and California)	0.2–0.6	SBS or EVA polymers (15 wt.%)	Study asphalt mixtures prepared from PPA/polymer-modified bitumen	Pen; SP; SARA; rheological parameters; ageing (RTFO, PAV); moisture resistance	(i) PPA enables the reduction of polymer content without loss of performance properties (ii) Improved processing conditions, high temperature viscosity, and storage stability	[43]

2005	Bitumen (Saudi Arabia, Venezuela)	0.6–1.2	—	Study the mechanisms for PPA modification	SARA; MW; ^31^P NMR; morphology (AFM)	(i) PPA stiffens the matrix (maltenes) or the dispersed phase (asphaltenes) depending on bitumen composition (i.e., source) (ii) Conversion of saturates into asphaltenes (Saudi Arabia) or resins into saturates and asphaltenes (Venezuela) (iii) Formation of asphaltene-phosphorous complexes	[44]

2006	Bitumen (Venezuela, Middle East)	0.4–1	—	Study the low-temperature performance (−25°C to +5°C)	Pen; SP; FraassT; rheological and thermal parameters	(i) PPA showed some positive effects on the low-temperature rheological behavior, and this influence depends mainly on the bitumen composition (i.e., source) (ii) PPA lowers the stiffness (at −25°C) and has marginal effects on ductility (at +5°C)	[22]

2007	Bitumen (Venezuela, Middle East)	0.4–1	—	Study high/medium temperature performance (+5°C to +100°C)	Pen; SP; ageing (RTFO, PAV); rheological and thermal parameters; FTIR	(i) PPA has a positive effect on the rheological behavior, providing the highest stiffening effects in the range 25 to 90°C (ii) The effect of PPA on the rheological properties depends mainly on the bitumen composition (i.e., source)	[45]

2008	Bitumen model compounds (isoquinoline; 1-methyl-2-quinolone)	16.7	—	Study the reaction with pyridine and pyridinone groups	FTIR	(i) Pyridine and pyridinone functional groups form ion pairs with PPA through hydration or by resonance, depending on the dielectric constant (ii) The dissociation and reaction of PPA with bitumen will occur preferentially in enclaves of high dielectric constant	[7]

2008	Bitumen model compounds (sulfur; tetrahydrothiophene; benzothiophene; tetramethylene sulfoxide)	16.7	—	Study the reaction with sulfide and sulfoxide groups	FTIR; TLC	(i) Aliphatic and aromatic sulfide groups were inert, at 150°C, thus invalidating the hypothesis of PPA induced nucleophilic displacement reactions (ii) Sulfoxide groups were very reactive with PPA suggesting that oxidized and nonoxidized bitumen will react differently	[15]

2008	Bitumen model compounds (indole)	3.2 and 16.7	—	Study the reaction with pyrrole functional groups	FTIR	(i) The amine and double bond of indole are both reactive (ii) The amine group undergoes phosphorylation (iii) N–N bridging increases MW and stiffness (iv) Disruption of the hydrogen bond network (involving pyrrole N–H groups) affects the asphaltenes-maltenes equilibrium (v) Competing bridging and ring opening reactions explain the increase/decrease of stiffness in PPA-modified bitumen	[46]

2008	Bitumen model compounds (bisphenol A; butyl phenyl ether; acetophenone; benzoic acid)	16.7	—	Study the reaction with oxygenated functional groups	FTIR; TLC	(i) The reactivity of the functional groups follows the order phenols > ketones > carboxylic acids > ethers (ii) Bisphenol A was fragmented (i.e., lower MW) whereas the other compounds were condensed into higher MW structures (iii) Phosphorylation of hydroxyl groups did not occur, showing that phosphate esters cannot exist in PPA-modified bitumen	[47]

2008	Bitumen ABD (SHRP library)^c^	1.5	SBS or Elvaloy® polymers (3 wt.%)	Study the reactions between PPA and bitumen or polymer-modified bitumen	^31^P NMR; storage stability	(i) PPA hydrolyzes back to orthophosphoric acid by reacting with residual water eventually present in bitumen (ii) The formation of organophosphate esters was not detected (iii) There is no evidence of reaction between SBS or Elvaloy polymers and bitumen in the presence of PPA (0.2 wt.%)	[32]

2008	AAD-1, AAM-1, and ABD bitumen (SHRP library)^c^	1.5	—	Study the variation of the rheological and chemical properties of PPA-modified asphalts with ageing time	Rheological and thermal parameters; FTIR; ^31^P NMR; ageing (PAV); storage stability	(i) PPA addition improves bitumen rheological properties (i.e., higher stiffness and greater elastic modulus) without significant changes in bitumen composition (ii) The effect of PPA depends on the chemical composition of the base bitumen (i.e., its source) (iii) Formation of organic phosphate esters was not observed	[20]

2009	AAA, AAU, AAX, and ABD bitumen (SHRP library)^c^	0-1	—	Study the reaction between PPA and bitumen with known contents of organic functional groups	SARA; MW; morphology (AFM); thermal parameters	(i) PPA acts at the interface between asphaltenes and maltenes, with resins (polar aromatics) playing a crucial role in the disruption of the hydrogen bond network (ii) PPA acts on functional groups with high dielectric constants (i.e., compounds with nitrogen, oxygen, or sulfur) (iii) The hydroxyl groups are not phosphorylated (iv) Tg of maltenes decreases and Tg of asphaltenes increases (v) High temperature performance grade (PG) correlates linearly with PPA content, depending on bitumen composition	[48]

2010	Bitumen (Repsol, Spain)	2	Crumb tire rubber (10 wt.%)	Study the properties of crumb rubber modified bitumen	Pen; SP; rheological and thermal parameters; storage stability; insoluble solids	(i) Small quantities of PPA improve the elastic properties of bitumen, due to sol-gel transition; Tg was unaffected (ii) The stage at which PPA is added (before or after mixing with crumb rubber) apparently has no influence (iii) PPA improves storage stability but is not sufficient to avoid sedimentation of crumb rubber particles at high temperature	[41]

2010	Paving bitumen (Lanzhou, China)	1–3	SBR, sulfur (0–6 wt.%)	Study the properties of bitumen modified with SBR, sulfur, and PPA	Pen; SP; FTIR; ageing (RTFO); mechanical, thermal, and rheological parameters; morphology; storage stability	(i) PPA shifts the structure from sol to gel improving the high-temperature physical and rheological properties of bitumen (ii) The addition of SBR can reduce the unfavorable effect of PPA on the low-temperature ductility (iii) The addition of sulfur to PPA/SBR-modified bitumen improves the high-temperature rheological properties, with a small effect on thermal stability	[49]

2011	AAD, AAM, and ABD bitumen (SHRP library)^c^	1.5	Hydrated lime (10 wt.%); dolomitic and granite fillers (10–30 wt.%)	Study the effect of hydrated lime and other fillers on the rheological and chemical properties of PPA-modified bitumen	Ageing (RTFO, PAV); ^31^P NMR; rheological parameters	(i) PPA increases bitumen stiffness (ii) The addition of granite fillers to PPA-modified bitumen has little effect on stiffness whereas dolomite reduces stiffness (iii) The addition of hydrated lime cancels the stiffening effect of PPA but slows down the oxidative ageing process of bitumen (iv) Hydrated lime reacts with PPA yielding calcium phosphates (v) The interaction between hydrated lime, PPA, and the binders is different for sol-type and gel-type bitumen	[50]

2011	PG58-22 bitumen (Iran)	1-2	Crumb rubber (5–15 wt.%); Vestnamer® (4.5 wt.% of crumb rubber)	Study the effect of PPA + Vestnamer (synthetic rubber) on crumb rubber modified bitumen	Pen; SP; ageing (RTFO, PAV); morphology; rheological parameters	(i) SP increases and Pen decreases linearly with PPA content (ii) The preferred content of PPA is 1 wt.%, since bitumen performance is improved both at high and at low temperatures (iii) Vestnamer reacts chemically with crumb rubber and bitumen to create a uniform macropolymer network (iv) The addition of PPA promotes the uniform distribution of crumb rubber and synthetic rubber particles in the bitumen, thus improving its rheological properties	[17]

2012	Bitumen (Russia, Saudi Arabia, and Venezuela)	0.5–1.5	—	Study the effect of PPA on bitumen behavior at low temperature (<5°C)	Pen; SP; FraassT; asphaltenes; wax content; rheological and thermal parameters	(i) PPA improves the low-temperature performance of bitumen (i.e., stiffness increases and Tg is lowered) up to 1 wt.% (ii) The effect of PPA at low temperature is strongly dependent on bitumen composition (i.e., wax and asphaltene content)	[21]

2012	Straight-run bitumen (Venezuela)	1-2	—	Study the molecular structure and mechanical behavior of PPA-modified bitumen	^1^H-NMR spin-spin relaxation times; rheological parameters	(i) 1% of PPA shifts the sol-gel transition temperature to higher values without bitumen loss of stability, whereas 2% of PPA imparts undesired heterogeneous structures (ii) The distribution of proton relaxation times provides useful information about the macrostructure of bitumen	[51]

2013	Bitumen (Saudi Arabia, China)	0.5–2	—	Study the effect of PPA on chemical composition, physical properties, and morphology of bitumen	Pen; SP; ductility; viscosity; SARA fractions; morphology (AFM)	(i) PPA apparently converts the resins into asphaltenes (ii) The effect of PPA depends on the type of bitumen, having a lower influence on bitumen with higher contents of resins and lower asphaltenes content (iii) In general, viscosity and SP increase with PPA content whereas Pen and ductility decrease (iv) Morphology and chemical composition of PPA-modified bitumen correlates strongly with the colloidal index	[32]

2013	Bitumen (Russia, Saudi Arabia, and Venezuela)	0.5–1.5	—	Study the changes induced by PPA in bitumen to relate its rheological properties and morphology	Pen; SP; morphology (SEM); rheological parameters	(i) PPA improves the high-temperature properties (i.e., extends the range of viscoelastic behavior), mainly due to the size reduction of asphaltenes micellar aggregates which improves solvation phenomena and colloidal stability (ii) The transition temperature from sol to gel increases with PPA concentration up to 1 wt.% and not so much for higher concentrations (depending on asphaltene content) suggesting the existence of saturation effects	[2]

2014	Bitumen (USA)	0.5	SBS; PPMA oxidized PE (2-3 wt.%); crumb rubber (10 wt.%)	Study the high temperature rheological properties of polymer-modified bitumen	Rheological parameters; ageing (RTFO, PAV)	(i) The addition of PPA (0.5 wt.%) can reduce polymer loading (from 3% to 2%) required to obtain the same bitumen grade (ii) The rheology of modified bitumen is source-dependent (iii) Oxidized PE and PPMA provided bitumen with lower viscosity values compared to SBS and crumb rubber, thus needing lower energy for mixing/compaction of the mixtures	[52]

2014	Bitumen (Petrobras, Brazil)	0.5–1.2	LDPE (3–6 wt.%)	Study high/medium temperature performance of LDPE-modified bitumen	Rheological parameters; ageing (RTFO, PAV)	(i) PPA improves the rheological properties (rutting and fatigue resistance) of the base bitumen, retaining performance grade (ii) Addition of PPA alone provides better improvements than modification with LDPE (with and without PPA)	[53]

^a^EVA: ethylene vinyl acetate, HDPE: high density polyethylene, LDPE: low density polyethylene, PE: polyethylene, PPMA: polypropylene-maleic anhydride, SBR: styrene-butadiene rubber, and SBS: styrene-butadiene-styrene block copolymers.

^b^AFM: atomic force microscopy, FraassT: Fraass breaking point temperature, FTIR: Fourier transform infrared spectroscopy, GPC: gel-permeation chromatography, MW: molecular weight, NMR: nuclear magnetic resonance spectroscopy, PAV: pressure ageing vessel test (long-term ageing ASTM D6521), Pen: penetration depth; RTFO: rolling thin film oven test (short-term ageing ASTM D2872), SARA: analysis of saturates, aromatics, resins, and asphaltenes, SEM: scanning electron microscopy, SP: softening temperature (ring and ball method), Tg: glass transition temperature, and TLC: thin-layer chromatography.

^c^SHRP: Strategic Highway Research Program.

## Appendix

This appendix presents a literature review concerning bitumen modification with polyphosphoric acid (PPA).  Table 6 lists the patents issued since 1973 when Alexander [55] patented a process using PPA to improve bitumen viscosity.  Table 7 summarizes the scientific studies performed to evaluate the effect of PPA on bitumen, or bitumen model compounds, and the main characteristics of the ensuing PPA-modified bitumen.
